# Gut feeling: Microbes at the heart of cancer therapy

**DOI:** 10.18632/oncotarget.28919

**Published:** 2026-09-23

**Authors:** Uzma Saqib, Sakina Ratlamwala, Monika Pandey, Krishnan Hajela

**Keywords:** gut microbiome, immunomodulation, precision therapeutics, tumor

## Abstract

The microbiome is the collection of microbes in a specific space. The human gut microbiome has emerging applications in cancer therapy, as it can modulate the immune system, enhance drug efficacy, and reduce tumorigenicity by various mechanisms. The gut microbiome can be exploited for the precision medicine in cancer therapeutics which gives a new direction to cancer therapy. The varied composition of the gut microbiome, a lack of standard procedures, and causalities could be the hurdles in this emerging therapy. However, it could be a promising therapeutic partner which could lead to new era of microbiome-based cancer therapy.

The current viewpoint focusses on the *role of the human microbiome in cancer*. The microbiome, comprising a diverse array of microorganisms residing in and on our bodies, has emerged as a critical determinant of human health. Recent research has demonstrated a crucial role in cancer development, progression, and therapeutic response, opening a new era in precision oncology. Scientific perspectives on cancer prevention, diagnosis, and treatment have been rapidly changing by dwelling deep into the interactions between host and microbiome (Figure 1).

**Figure 1:**
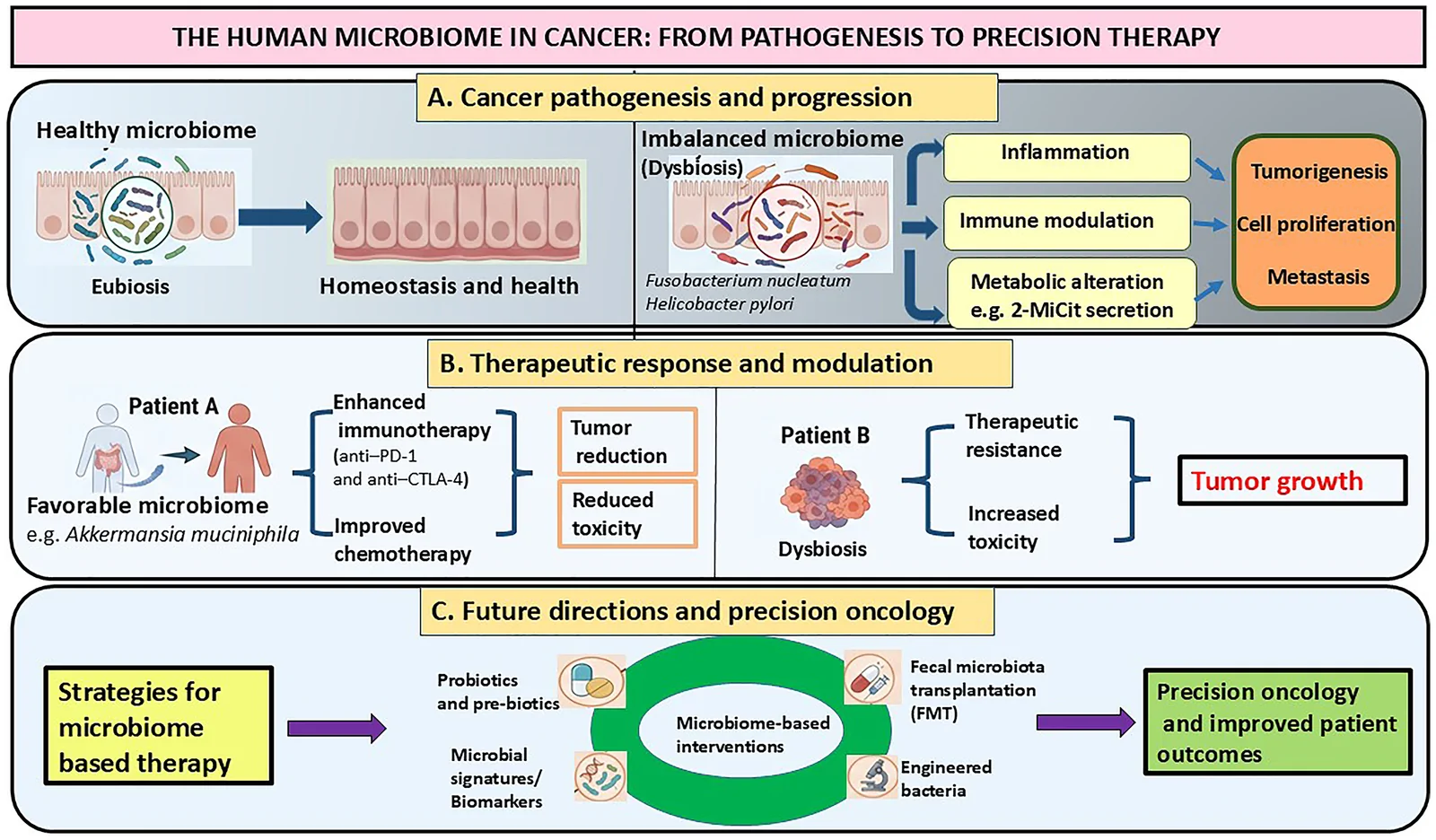
Overview of the human microbiome in cancer: from pathogenesis to precision therapy. (**A**) Cancer pathogenesis and progression: A healthy microbiome or eubiosis supports the intestinal lining leading to health and homeostasis whereas dysbiosis promotes cancer through mechanisms like inflammation, immune modulation and metabolism alteration that triggers tumorigenesis, cell proliferation and metastasis. (**B**) Therapeutic response and modulation: Favorable microbiome enhances immunotherapy (boosts the effectiveness of check point inhibitors like anti-PD-1 and anti-CTLA-4) and chemotherapy efficacy, reduces tumor and toxicity, while dysbiosis increases therapeutic resistance and toxicity leading to tumor growth and treatment failure. (**C**) Future directions and precision oncology: Future therapeutic strategies including probiotics, prebiotics to restore healthy microbiome balance, fecal microbiota transplantation from healthy donor to re-seed patient’s microbiome, using synthetic biology to engineer bacteria that can target tumor and using microbiome biomarkers to predict specific treatment for the patient. These strategies leading to precision oncology can provide improved patient outcomes.

The gut microbiome influences cancer risk through mechanisms involving inflammation, immune modulation, and metabolism [1–3]. Dysbiosis, or microbial imbalance, has been associated with multiple cancers. Certain bacterial species secrete metabolites that promote tumorigenesis or reshape the tumor microenvironment, thereby enhancing cancer cell proliferation and survival [4]. For instance, *Fusobacterium nucleatum* promotes colorectal cancer by suppressing immune activity and activating oncogenic signaling [5], while *Helicobacter pylori* induces gastric cancer through chronic inflammation and DNA damage [6]. Beyond localized effects, microbial metabolites such as short-chain fatty acids and bile acids regulate systemic metabolism, epigenetic mechanisms, and immune surveillance, revealing the intricate interplay of host–microbe–tumor interactions [7].

Emerging evidence also underscores the microbiome’s influence on cancer therapy efficacy and toxicity [8]. Responses to immune checkpoint inhibitors, including anti–PD-1 and anti–CTLA-4 therapies, are enhanced in patients with diverse and beneficial gut microbiota that promote dendritic cell maturation, T-cell priming, and anti-tumor immunity [9–11]. Conversely, dysbiosis can induce therapeutic resistance. Similarly, chemotherapy outcomes are affected by microbial metabolism, which can activate or inactivate drugs and modulate gastrointestinal toxicity [12]. These insights highlight the potential of microbiome modulation to improve treatment effectiveness while minimizing adverse effects.

A major breakthrough has been the identification of microbial signatures predictive of immunotherapy response [13]. Supplementation with beneficial microbes such as *Akkermansia muciniphila* has been shown to boost anti–PD-1 efficacy in preclinical models [14], inspiring clinical trials exploring probiotics, prebiotics, and fecal microbiota transplantation to enhance therapy outcomes [14]. Beyond treatment, microbiome profiling offers promise as a predictive biomarker for therapy selection and monitoring patient response.

Adding to this growing evidence, scientists recently discovered that bacteria residing within tumors can secrete 2-methylisocitrate (2-MiCit), a metabolite that sensitizes tumor cells to chemotherapy. This bacterial molecule disrupts DNA repair and metabolic balance, thereby amplifying the cytotoxic effects of standard chemotherapeutics such as 5-fluorouracil in colorectal cancer models. Daniel et al. showed that 2-MiCit exhibits anti -proliferative properties and reduces tumor size in Drosophila gut tumor model. 2-Methylisocitrate (2-MiCit) sensitizes tumor cells by simultaneously disrupting mitochondrial metabolism and inducing DNA damage [15]. It inhibits mitochondrial isocitrate dehydrogenase (IDH), a key TCA-cycle enzyme, which impairs oxidative metabolism and ATP production while altering the supply of biosynthetic intermediates. This metabolic blockade creates energy stress and, critically, a nucleotide imbalance that destabilizes DNA replication and repair. Multi-omic analyses show that 2-MiCit activates DNA-damage response pathways, indicating that the nucleotide pool disruption leads to DNA lesions. The resulting combination of energy stress and DNA damage lowers the threshold for chemotherapy-induced cell death, explaining its synergy with agents like 5-fluorouracil [16].

Furthermore, introducing a defined mix of non-pathogenic intratumoral bacteria, like *Proteus mirabilis* and *Rhodopseudomonas palustris*, has been shown to reduce tumors, even in immunocompromised models, without systemic toxicity [17]. There are two mechanisms by which this bacterial consortium shows tumor regression; primary mechanism involves tumor necrosis through vascular collapse and thrombosis [18], while secondary mechanism involves intratumoral biofilm formation and metabolic stress like iron sequestration using siderophores [19].

An emerging dimension of the microbiome- cancer interaction involves microbial extracellular vesicles (mEVs), including bacterial Outer Membrane Vesicles (OMVs). Bacterial OMVs are tiny vesicles secreted by Gram-negative bacteria, that have caught researchers attention due to its distinctive characteristics which helps in drug delivery, immune system modulation with minimal harm to healthy cells and less systemic toxicity [20]. They function as vehicles for the selective transport of bioactive cargo, including proteins, lipids, nucleic acids, metabolites and virulence associated factors potentially targeting host immune response, influencing immune signals, cytokine production and the tumor microenvironment, thereby facilitating inter-kingdom communication and drug resistance [21, 22].

Microbial extracellular vesicles (mEVs), small membrane-bound particles naturally released by microorganisms for communication, virulence, and immune modulation, may exert both pro- and anti-tumor effects [23]. In cancer, these vesicles are emerging versatile therapeutic tools as they favor anti- tumor responses by reprogramming immune cells and cytokine networks [24]. They can potentially deliver chemotherapeutics and act as immunostimulants [25]. Extracellular vesicles derived from *Fusobacterium nucleatum*, have shown its presence in experimental model of colorectal cancer tissues, it facilitated bacterial adhesion and intratumoral colonization, promoting tumor progression [26]. Preclinical studies in colorectal, melanoma, lungs, and other cancers show that engineered or commensal-derived mEVs can suppress tumor growth, enhance T-cell infiltration, overcome drug resistance, and improve survival, often synergizing with chemotherapy or immunotherapy. In particular, bacterial OMVs can activate cancer-antigen-specific CD8+ T-cell responses and have demonstrated synergistic activity with anti-PD1 immunotherapy in preclinical models. Larger-scale production of OMVs for cancer therapy is the biggest hurdle which was overcome by purifying outer membrane proteins from bacteria and in vitro synthesis of OMVs, which shows effective treatment on tumors along with anti PD- 1 immunotherapy [27]. These findings suggest that engineered mEVs can influence cancer biology and emerge as an important tools for regulating therapeutic responses. In parallel, the presence of microbial EVs and their bioactive cargo in accessible biological fluids has made a possibility of making EV-based biomarkers for the detection of cancer, monitoring disease and also treatment response prediction, making it a valuable tool for targeted drug delivery and cancer immunotherapy. Microbial EVs are also being explored for liquid-biopsy application as potential biomarker of cancer, with stool microbial EV showing potential for diagnosis of colorectal cancer [28, 29]. However, clinical translation faces challenges in standardizing isolation methods, ensuring safety by controlling immunogenicity and endotoxin content, optimizing tumor targeting, and establishing regulatory frameworks, making microbial EVs a promising but still evolving frontier in microbiome-based cancer therapy.

## Limitations and future directions

Despite significant advances in the field, several translational and methodological challenges represent a limitation for the clinical application of human microbiome-based approaches in cancer management. The composition of the microbiome is strongly influenced by host genetics, lifestyle, geographical conditions, diet, medication use, disease stage and tumor type. Additionally, variation in sample collection, extraction of DNA, sequencing methods, and bioinformatics pipelines also contribute to inconsistent findings in studies, highlighting the need for standardized methodologies [30].

Establishing causality is another major hurdle. Although microbial dysbiosis has been implicated in tumor initiation, progression and therapeutic response, it is often hard to figure out whether these microbial alterations are causal drivers of carcinogenesis, secondary consequences of tumor associated changes, or just simply a bystander. Even though, germ free and gnotobiotic models have provided mechanistic insights, applying those results directly to human health is still very hard and limited [31–33]. Furthermore, substantial inter-individual variability in the composition of the microbiome results in mixed responses to microbiome-based strategies, including prebiotics, probiotics and fecal microbiota transplantation, underscoring the need for personalized therapeutic strategies [34, 35]. While most studies have focused on the gut microbiome due to its main role in systemic immunity and metabolism, growing evidence conclude that the microorganisms residing directly within tumor are equally contributing and playing active roles in regulating tumor biology and therapeutic response. Although, both fields face technical limitations. Study of intratumoral microbiome is also very tedious due to low availability of microbial biomass and its susceptibility to contamination, thus necessitating rigorous quality control.

Future research should focus on standardizing protocols, multicentre longitudinal studies, focusing on multi-omics approach for establishing causality and also validating microbiome derived biomarkers. Patient-specific microbiome profiling combined with engineered microbial therapeutics, may clear the path for the development of safe and effective strategies for precision oncology [36].

Collectively, these discoveries redefine the microbiome as an active therapeutic partner rather than a passive bystander. Harnessing microbial metabolites, engineered bacteria, and microbiome signatures could lead to a new era of microbiome-based cancer therapy. This is expected to advance precision oncology by enhancing efficacy, reducing toxicity, and augmenting therapeutic outcomes.
